# Two genera of Mymaridae (Hymenoptera) new to Africa, a remarkable new species of *Anaphes* and new generic synonymy

**DOI:** 10.3897/zookeys.658.11569

**Published:** 2017-02-22

**Authors:** John T. Huber, Serguei V. Triapitsyn

**Affiliations:** 1 Natural Resources Canada c/o AAFC, 960 Carling Ave., Ottawa, ON, K1A 0C6, Canada; 2 Entomology Research Museum, Department of Entomology, University of California, Riverside, CA, 92521, USA

**Keywords:** Mymaridae, *Paranaphoidea*, *Cleruchus*, *Allanagrus*, central Africa, taxonomy

## Abstract

*Bakkendorfia* Mathot, **syn. n.** is placed in synonymy under *Cleruchus* Enock and its only described species transferred as *Cleruchus musangae* (Mathot), **comb. n.** *Anaphes quinquearticulatus* Huber & Triapitsyn, **sp. n.** is described; it is the only known *Anaphes* Haliday species with a 5-segmented funicle in females. Two genera are reported for the first time from the Afrotropical region and two species are described: Paranaphoidea (Idiocentrus) africana Huber & Triapitsyn, **sp. n.**, and *Allanagrus occidentalis* Huber & Triapitsyn, **sp. n**.

## Introduction

While studying specimens representing many species of Mymaridae (Hymenoptera: Chalcidoidea) from the Afrotropical region in preparation for an illustrated identification key to the genera occurring in the region we discovered that *Bakkendorfia* Mathot is an unrecognized junior synonym, which we place in the proper synonymy. A new species of *Anaphes* Haliday is also described because it has some remarkable unusual features that expand the generic definition of the genus. Finally, we also describe one new species in each of the two genera not previously reported for the region: *Paranaphoidea* Girault and *Allanagrus* Noyes & Valentine.

## Methods

Specimens of the new species were all slide mounted in Canada balsam. Absolute measurements are given in micrometers, converted from filar micrometer eyepiece measurements. However, ratios for the body parts of each specimen were first determined from filar eyepiece micrometer measurements of length and width in order to find the minimum and maximum ratio for each antennal segment. The measurements were then converted to micrometers. Because of rounding errors, it appears that the ratios are slightly incorrect compared to those that are calculated using the absolute measurements (micrometers) but, in fact, they are more accurate and are therefore given in the species descriptions. Specimens are deposited in the Natural History Museum, London, England, UK (**BMNH**), the Canadian National Collection of Insects, Arachnids and Nematodes, Ottawa, Ontario, Canada (**CNC**), and the Entomology Research Museum, University of California, Riverside, California, USA (**UCRC**). Photographs were taken with a ProgRes C14^plus^ digital camera attached to a Nikon Eclipse E800 compound microscope, and a selection of the resulting layers combined electronically in Zerene Stacker™. Abbreviations used in the descriptions are: **fl_x_** for funicle segment, **gt_x_** for gastral tergum and mps for multiporous plate sensilla.

## Taxonomy

### Cleruchus

Taxon classificationAnimaliaHymenopteraMymaridae

Enock, 1909

Bakkendorfia Mathot, 1966. **Syn. n.**
Douttiella Annecke, 1961. Synonymy under Cleruchus by Noyes & Valentine, 1989: 31.
Eucleruchus Ogloblin, 1940. Synonymy under Cleruchus by Luft Albarracin et al., 2009: 26.
Haplochaeta Noyes & Valentine, 1989. Synonymy under Cleruchus by Lin et al., 2007: 29.
Paracleruchus Yoshimoto, 1971. Synonymy under Cleruchus by Viggiani, 1974: 88.
Stenopteromymar Ferrière, 1952. Synonymy under Cleruchus by Viggiani, 1974: 88.

#### Type species.

*Cleruchus pluteus* Enock.

The worldwide genus *Cleruchus* contains a variety of species known as parasitoids of Coleoptera (Triapitsyn et al. 2013, Barnes 2014). *Bakkendorfia* contains only one described species, *Bakkendorfia musangae* Mathot that Mathot (1966) treated as being related to *Parallelaptera* Enock (now a subgenus of *Erythmelus* Enock) based on a large hypopygium. While this is one defining feature of *Erythmelus*, Mathot’s species differs from *Erythmelus* in many other respects, particularly in the structure of the head and mandibles.

**Figures 1–3.**
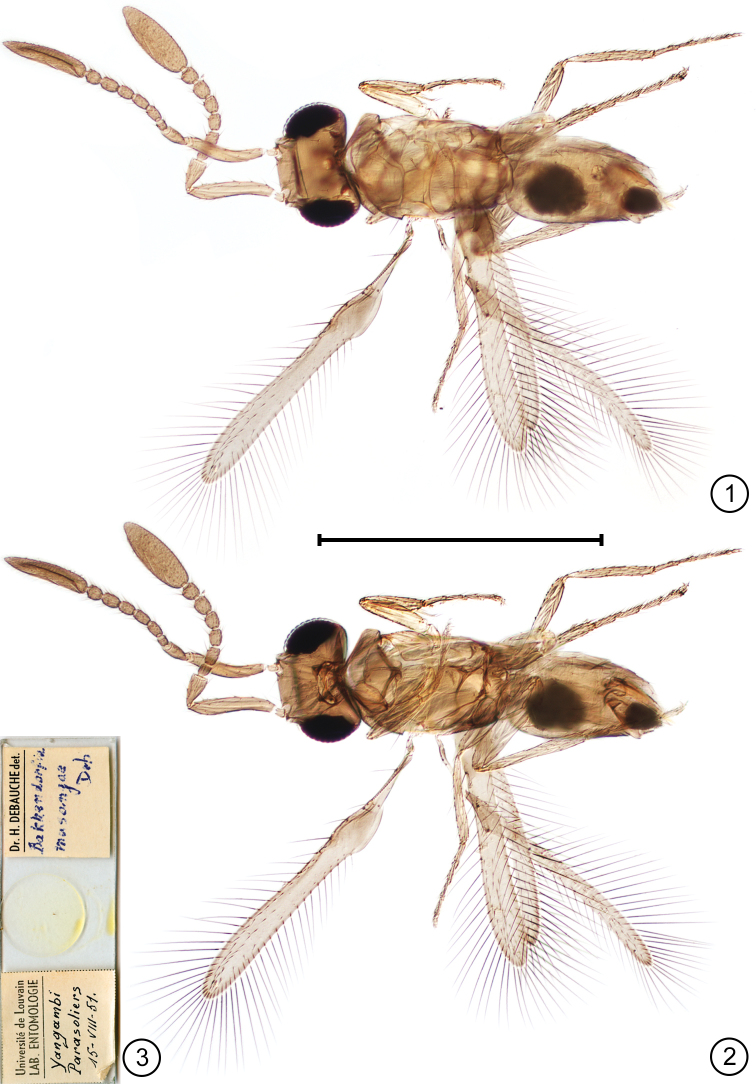
*Cleruchus musangae* (Mathot), holotype habitus **1** dorsal **2** ventral, as seen through body from above **3** holotype slide. Scale bar: 500 μm.

**Figures 4, 5.**
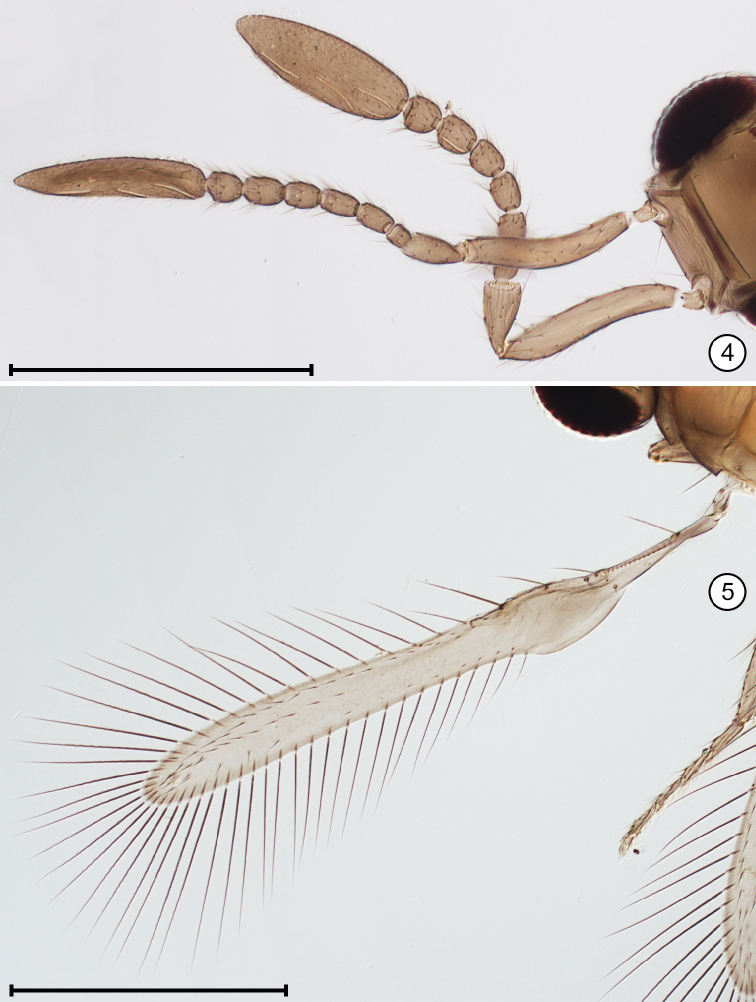
*Cleruchus musangae* (Mathot), holotype **4** antennae **5** fore wing. Scale bars: 200 μm.

**Figure 6.**
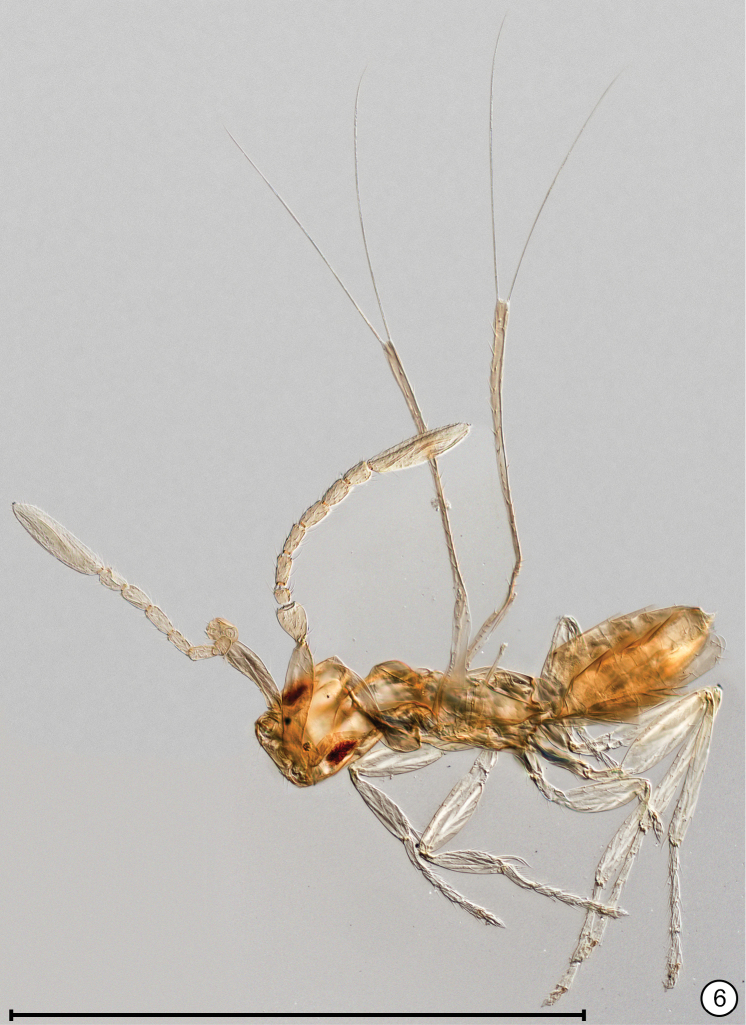
*Cleruchus biciliatus* (Ferrière), paratype habitus. Scale bar: 500 μm.

We examined the type series (Fig. 3, holotype slide) and found that *Bakkendorfia* matches *Cleruchus* in all its features. We therefore transfer the type species to *Cleruchus* as *Cleruchus musangae* (Mathot), comb. n., and illustrate it. Features that define *Cleruchus* include: posterior ocelli widely separated and close to the eye margins, forming a low ocellar triangle (Fig. 1); frenum transverse and somewhat oval in shape (Fig. 1); head in lateral view with a distinctly bulging face; and mandible with 2 teeth. Females have a short ovipositor (Fig. 2) and large clava almost as long as the relatively short funicle, the individual segments of which are usually more or less quadrate (Figs 1, 2, 4). The most obvious generic feature, a parallel-sided fore wing with few microtrichia on its surface and a slightly widened and curved stigmal vein (Fig. 5), is one that is not always present because the degree of wing development varies considerably among species of *Cleruchus*. Wingless species have been described (Yoshimoto 1971, Triapitsyn et al. 2013, Triapitsyn 2014a) and among fully winged species, the fore wing of *Cleruchus biciliatus* (Ferrière) has a greatly reduced surface and only two (or sometimes four) long marginal setae and the hind wing is greatly shortened, without marginal setae (Fig. 6).

### Anaphes

Taxon classificationAnimaliaHymenopteraMymaridae

Haliday, 1833

Extensive synonymy given in Huber (1992).

#### Type species.

A formal decision by ICZN on the correct type species to use is pending so the species in not named here. See petition by Huber et al. (2011) and comments and corrigendum (Huber 2014).

The worldwide genus *Anaphes* contains a variety of species known as parasitoids of several insect orders, summarized in Huber (1986). Features that define *Anaphes* include: fore wing with socketed seta present at apex of frenal fold; propodeum with a median longitudinal groove; petiole short, almost vertical, much wider than long crescent closely appressed to gt_1_; and gt_1_ longitudinally divided medially.

### Anaphes quinquearticulatus

Taxon classificationAnimaliaHymenopteraMymaridae

Huber & Triapitsyn sp. n.

http://zoobank.org/28FA1177-48E1-4F82-A67A-36B01C59705D

Figs 7–8 , 9, 10 , 11–13 , 14–16

#### Type material.

Holotype female (UCRC) on slide (Fig. 16) labelled: 1. “Anaphes quinquearticulatus Huber & Triapitsyn ♀ dorsal Holotype”. 2. “Rep. Congo: Pool Abio, Lesio-Louna Park, 3°6'1"S, 15°31'26"E 29.vii.2008 Sharkey MT”. 3.“Univ. Calif. Riverside Ent. Res. Museum UCRC Ent. 264592”. 4. “Mounted at UCR/ERM by V.V. Berezovskiy 2008 in Canada balsam”. Paratypes: 3 females. REPUBLIC OF THE CONGO. Pool. Lesio-Louna Wildlife Reserve, Abio, 330 m, 3°06.020'S, 15°31.440'E, 29.vii.2008, M. Sharkey & Y. Braet, MT (1 female, UCRC); Iboubikro, 3°16'11"S, 15°28'16"E, 23.vii.2008, M. Sharkey, MT (2 females, CNC, UCRC).

**Figures 7, 8.**
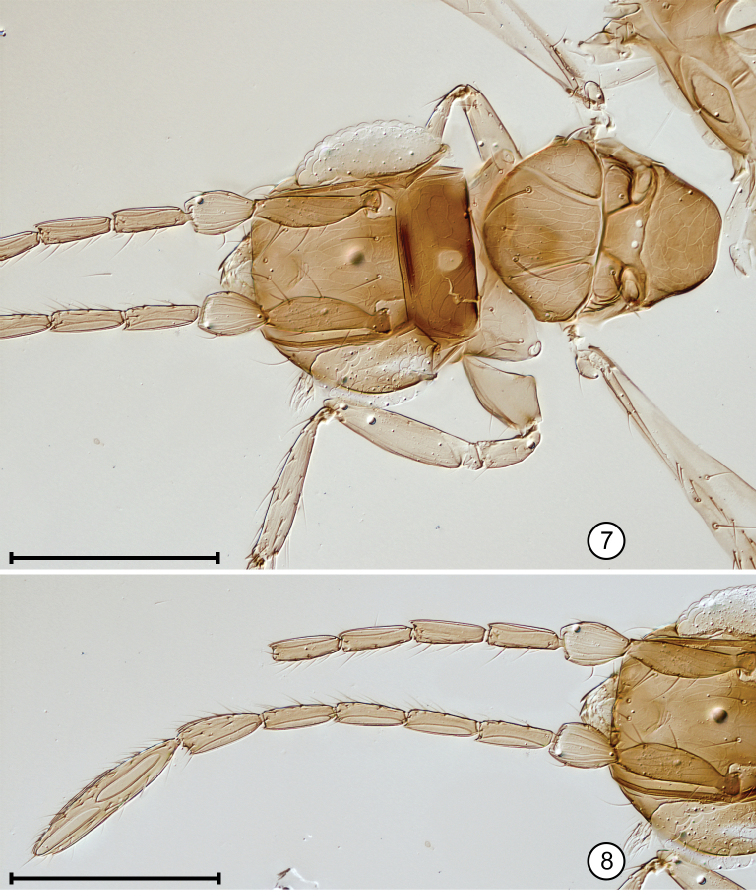
*Anaphes quinquearticulatus*, holotype **7** head + mesosoma **8** antennae. Scale bars: 100 μm.

#### Diagnosis.

**Figures 9, 10.**
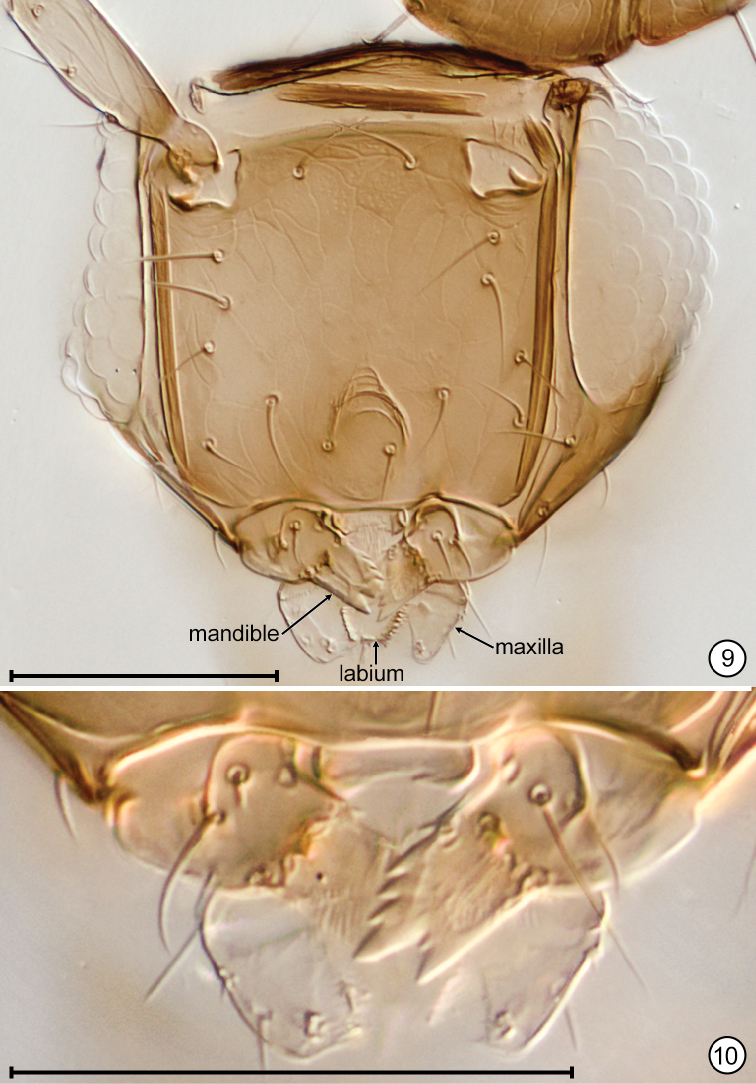
*Anaphes quinquearticulatus*, paratype **9** head, anterior showing other mandible **10** mouthparts. Scale bars: 50 μm.

**Female.** Funicle 5-segmented (Figs 8, 15), with 1 mps on fl_1_–fl_4_ and 2 mps on fl_5_ (Fig. 8), the mps unusually wide (Figs 7, 8). Mandible with 5 teeth (Figs 9, 10).

This species is unique among *Anaphes* in having only five funicle segments, instead of six as in all other described species. Otherwise, *Anaphes quinquearticulatus* has all the diagnostic features (listed above) of *Anaphes*. Because fl_1_ bears a distinct mps and is as long as any of the remaining segments it is almost certain that the first funicle segment in *Anaphes quinquearticulatus*, not some other segment, was lost, i.e., fl_1_ in this species is equivalent to fl_2_ in any other *Anaphes* species. This is because fl_1_ in females of all other *Anaphes* never have mps and is almost always distinctly shorter than fl_2_ or any other segment. The fore wing almost devoid of surface microtrichia is also unique; the single line of microtrichia present represents the line that separates the marginal from the medial spaces in any other *Anaphes* species. The narrow evenly curved fore wing is also interesting; it is most similar to undescribed *Anaphes* species found near water in the Oriental region.

**Figures 11–13.**
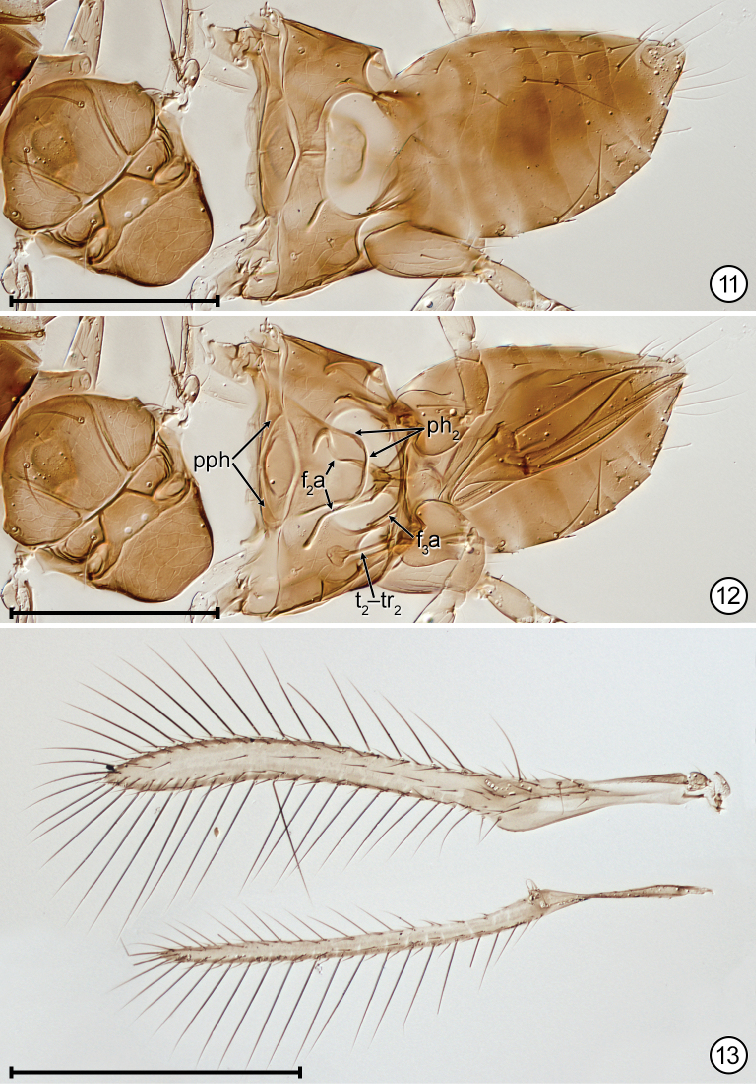
*Anaphes quinquearticulatus*. **11** holotype mesosoma + metasomadorsal **12** holotype mesosoma + metasoma, ventral as seen through body **13** paratype wings. Scale bars: 100 μm (**11, 12**) , 200 μm (**13**).

#### Description.

**Female.** Body length 255–358 (n=4, slide specimens) (dry length of one paratype before slide mounting 264). Dark brown (presumably), appendages apparently lighter in colour (cleared specimens so colour not really known). Fore wing margin in apical half narrowly but distinctly margined with brown, otherwise with faint uniform brown suffusion over most of surface except partly behind venation.

*Head*. Head width 123–135 (n=3). Face with 7 setae on each side (Fig. 9) and with faint reticulate sculpture. Mouthparts (Figs 9, 10) with mandible about as long as maxilla and with 5 teeth, the two ventral ones large, the 3 dorsal ones small and in one specimen a small tooth between the large ventral ones (Figs 9, 10). Occiput with vertexal suture (= supraorbital suture extension onto occiput) long and in line with supraorbital trabecula, only weakly diverging from posterior eye margin.

*Antenna*. Scape on inner surface and pedicel with longitudinally reticulate sculpture; funicle 5-segmented, with 1 mps on fl_1_–fl_4_ and 2 mps on fl_5_ (Fig. 8, 15), the mps unusually wide (Figs 7, 8); clava with 6 mps. Measurements (length/width, n = 3 or 4) of antennal segments: scape 53–60/12–16, pedicel 29–34/20–22, fl_1_ 35–37/11–12, fl_2_ 33–35/10–12, fl_3_ 32–34/10–11, fl_4_ 33–35/10–12, fl_5_ 38–42/13–16, clava 79–86/19–23. Length/width ratios of antennal segments: scape 3.29–4.26, pedicel 1.52–1.66, fl_1_ 3.10–3.41, fl_2_ 3.19–3.63, fl_3_ 3.04–3.44, fl_4_ 3.04–3.48, fl_5_ 2.60–2.79, clava ≈3.69–4.55 (clava not always oriented in perfect lateral view).

*Mesosoma*. Mesoscutum width 82–90 (n=3), with coarse reticulate sculpture, the cells irregularly shaped but more longitudinally stretched on midlobe, more isodiametric anteriorly on lateral lobe (Fig. 7); scutellum with coarse reticulate sculpture, the cells smaller on anterior scutellum, larger and more transversally stretched on frenum (Fig. 7); dorsellum apparently smooth (Figs 11, 12; propodeum with sculpture as on frenum (Figs 11, 12). Mesoscutal midlobe and axilla with relatively long setae.

*Wings*. Fore wing narrow, beyond level of venation with evenly concave posterior margin and surface with one row of about 10 microtrichia extending from stigmal vein almost to wing apex and a second row extending proximally from socketed seta at apex of frenal fold to just past base of parastigma (Fig. 13). Hind wing without microtrichia on surface between the usual anterior and posterior rows. Fore wing length (n=4) 394–428, width 30–33, length/width 13.0–13.5, longest marginal setae ≈106–127. Hind wing length 376–414, width 13–15, longest marginal setae 86–94.

**Figures 14–16.**
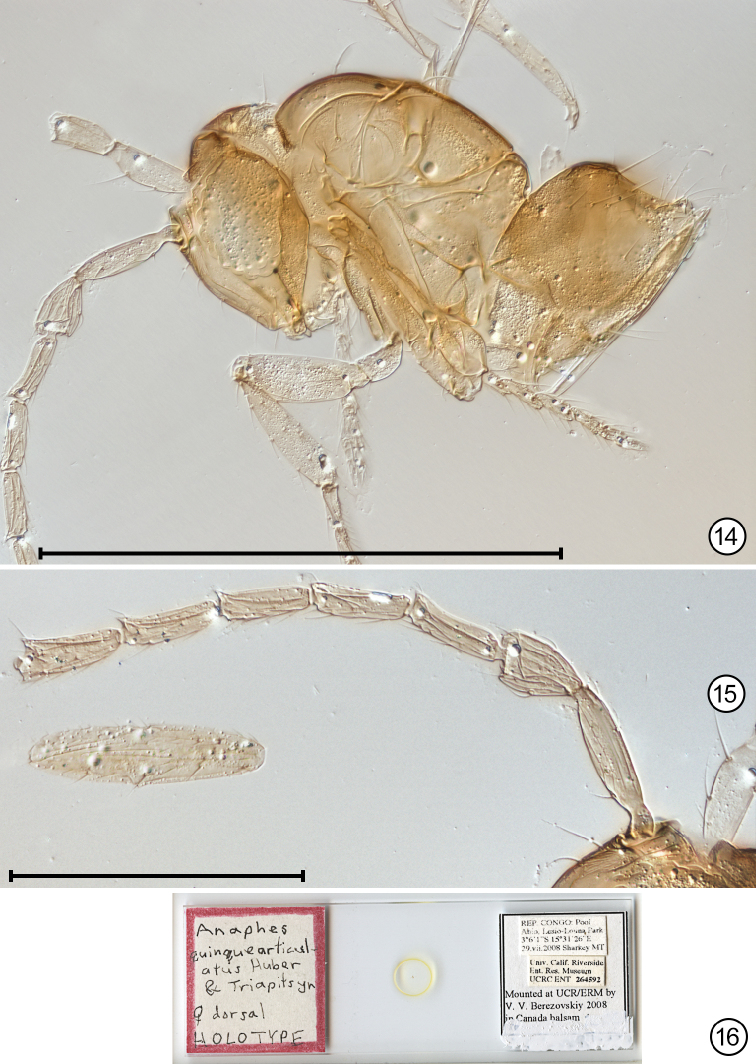
*Anaphes quinquearticulatus*. **14** paratype, lateral habitus **15** antenna **16** holotype slide. Scale bars: 500 μm (**14**) , 100 μm (**15**).

*Legs*. Metatarsomere 1 0.78–0.92 × as long as metatarsomere 2 (Fig. 14).

*Metasoma*. Ovipositor length 124–129 (n=4), 1.13–1.20 × as long as metatibia length (104–114) and extending slightly forward of junction between mesosoma and metasoma (Fig. 14, gaster slightly crushed and poorly oriented).

**Male**. Unknown.

#### Derivation of species name.

Latin for five + articulated; an adjective referring to the unique 5-segmented funicle, the first *Anaphes* to be described with this reduced antennal segmentation.

### Paranaphoidea

Taxon classificationAnimaliaHymenopteraMymaridae

Girault, 1913

Idiocentrus Gahan, 1927: 35. Proposed as a subgenus and synonymized under Paranaphoidea by Lin et al., 2007: 43.

#### Type species.

*Paranaphoidea egregia* Girault.

*Paranaphoidea* contains several described species in Australia (Noyes & Valentine 1989) and one in New Zealand, *Paranaphoidea mira* (Gahan), that was supposedly reared from *Melampsalta muta* Fabricius (Hemiptera: Cicadidae) (Gahan 1927). The species name of Paranaphoidea (Idiocentrus) mira, which was transferred to *Paranaphoidea* by an implied combination at the time of generic synonymy of *Idiocentrus* by Lin et al. (2007), is an adjective and consequently is being treated here to properly match the feminine gender of *Paranaphoidea*. Features that define *Paranaphoidea* include: clava 2- or 3-segmented; face with subantennal groove extending ventrally from each torulus; occiput with a transverse curved groove from eye to eye and medially above foramen; ovipositor projecting anteriorly under mesosoma; and frenum medially divided by a shallow longitudinal groove. The fore wing apex is truncate or rounded and the hind wing is relatively wide in most, but not all, Australian species compared to the rounded fore wing apex and relatively narrow hind wing in the New Zealand species. The ovipositor projects forward under the mesosoma to varying degrees in Australian species and beyond the front of the head in the New Zealand species and also in one undescribed Paranaphoidea (Idiocentrus) sp. from Western Australia (UCRC). The two subgenera are most easily separated by the number of segments in the clava, either two in Paranaphoidea (Paranaphoidea), as in all the Australian species described so far, or three in Paranaphoidea (Idiocentrus), as in the single described New Zealand species.

Even taking into account two specimens (CNC) of an unidentified species of Paranaphoidea (Idiocentrus) from Thailand, the presence of a species of Paranaphoidea (Idiocentrus) in West Africa represents a huge extension in range of *Paranaphoidea*. We thought perhaps that the African specimen was either mislabelled or was accidentally introduced but these possibilities seem unlikely. Other cases of wide ranges in representatives of a genus initially known to occur only in one region are not uncommon in Mymaridae, e.g., *Chrysoctonus* Mathot (Huber & Triapitsyn 2015), and intensive collecting eventually results in more specimens of different (or sometimes the same) species from intervening areas being discovered. We treat the specimen below as a new species even though it is extremely similar to *Paranaphoidea mira* from New Zealand. The recorded host of *Paranaphoidea mira*, *Melampsalta muta* is now in *Kikihia* Dugdale, all of whose species are endemic to New Zealand. It would be interesting to discover the host(s) of *Paranaphoidea* species that occur outside of New Zealand.

### Paranaphoidea (Idiocentrus) africana

Taxon classificationAnimaliaHymenopteraMymaridae

Huber & Triapitsyn sp. n.

http://zoobank.org/8D8E3FC0-1475-4C6F-A11A-36F3FF5ED373

Figs 17–19 , 20, 21 , 22–24

#### Type material.

**Figures 17–19.**
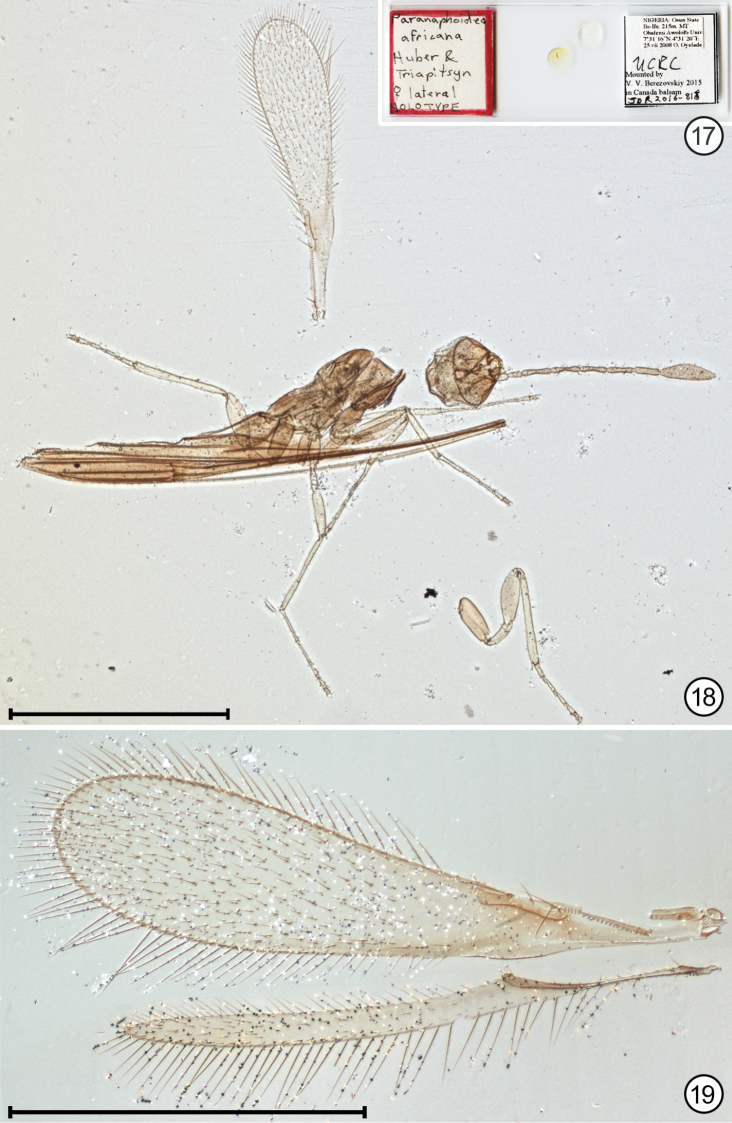
*Paranaphoidea africana* holotype **17** holotype slide **18** habitus **19** wings. Scale bars: 1000 μm (**18**), 500 μm (**19**).

Holotype female (UCRC) on slide (Fig. 17) labelled: 1. “Paranaphoidea africana Huber & Triapitsyn ♀ lateral Holotype”. 2. “Nigeria: Osun State Ile-Ife, 215m, MT Obafemi Awololo [sic] Univ. 7°31'16"N, 4°31'20"E”. 3. “UCRC Mounted by V.V. Berezovskiy 2015 in Canada balsam JDR 2016-818”.

#### Diagnosis.

**Female.** Clava 3-segmented (division between segment 1 and 2 faint), fl_1_ 1.54 × as long as fl_2_ and without mps (Fig. 24). *Paranaphoidea africana* differs in claval segmentation from the Australian species of Paranaphoidea (Paranaphoidea), all of which have a 2-segmented clava. It is most similar to Paranaphoidea (Idiocentrus) mira from New Zealand. It differs from a non-type specimen (in CNC) of *Paranaphoidea mira* by its fore wing wider (narrower in *Paranaphoidea mira*, with length/width = 4.88), the hind wing uniformly narrow from apex of venation to just before wing apex and length/width = 16.3 (hind wing slightly wider towards apex, with length/width = 13.7 in *Paranaphoidea mira*).

#### Description.

Mesosoma + metasoma length 1180 (head mounted face view so its length cannot be measured). Body brown (presumably) except frenum yellowish; upper half of occiput, pedicel, and perhaps also pronotum laterally, propodeum laterally and metapleuron lighter brown; legs except coxae lighter than body (cleared specimen so colour not really known) (Figs 18, 22, 23). Fore wing margin narrowly but distinctly margined with brown, otherwise with faint uniform brown tinge behind submarginal vein and parastigma (except narrowly immediately behind submarginal vein) and along proximal third of hind margin. Hind wing with apical half slightly suffused with brown (Fig. 19).

**Figures 20, 21.**
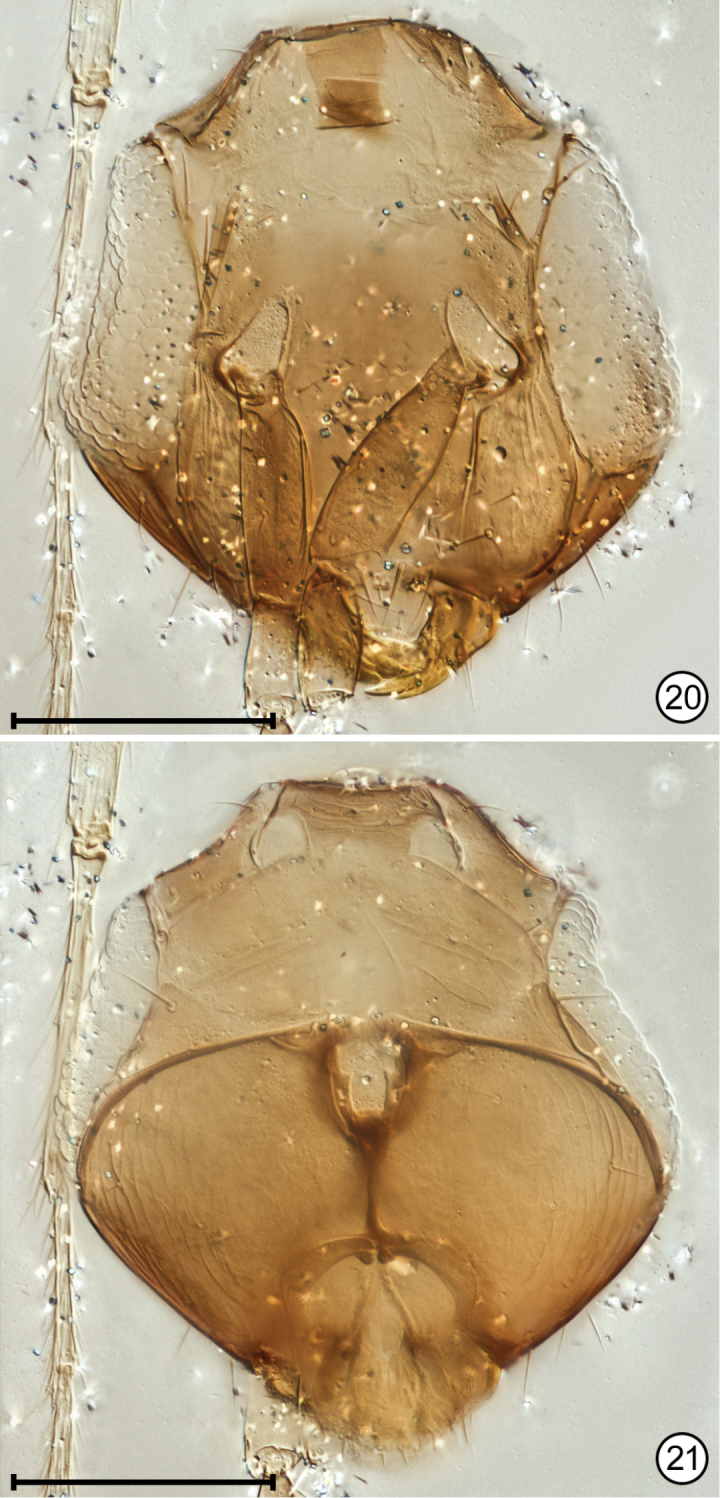
*Paranaphoidea africana* holotype **20** head, anterior **21** head, posterior as seen through head. Scale bars: 100 μm.

*Head*. Head width 236 (eyes collapsed so width should be slightly wider) (Figs 20, 21).

*Antenna*. Funicle without mps on fl_1_ and with 2 mps on fl_2_–fl_6_ (Fig. 24); clava with 7 mps, 2 each on segments 1 and 2, and 3 on segment 3 (Fig. 24). Measurements (length/width) of antennal segments: scape 100/30, pedicel 55/24, fl_1_ ≈50/18, fl_2_ 94/19, fl_3_ 93/18, fl_4_ 87/18, fl_5_ 86/18, fl_6_ 86/21, entire clava 181/50, with segments 1–3 (measured along dorsal margin) 40, 50, and 92, respectively. Length/width ratios of antennal segments: scape 3.33, pedicel 2.25, fl_1_ 2.84, fl_2_ 4.90, fl_3_ 5.04, fl_4_ 4.93, fl_5_ 4.88, fl_6_ 4.13, entire clava 3.65.

**Figures 22–24.**
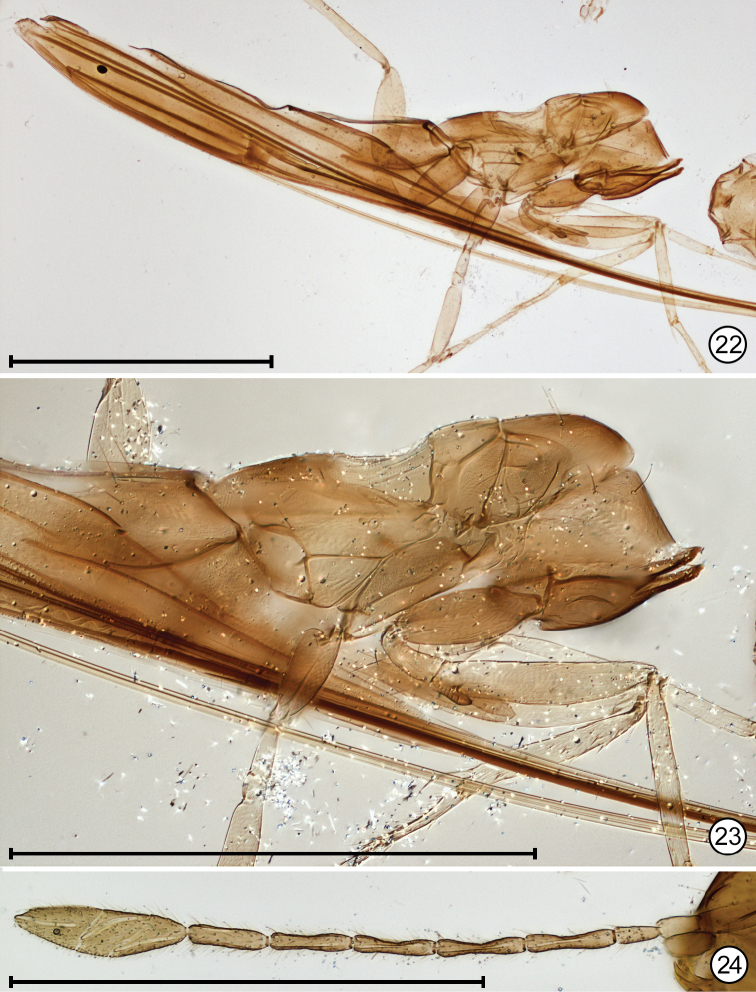
*Paranaphoidea africana* holotype **22** mesosoma + metasoma, lateral **23** mesosoma + base of metasoma, lateral **24** antenna. Scale bars: 500 μm.

*Mesosoma*. Frenum poorly sclerotized and collapsed inward (Figs 22, 23).

*Wings*. Fore wing with evenly rounded apex, without microtrichia behind and just beyond venation, then microtrichia evenly distributed to wing apex. Hind wing parallel-sided throughout its length beyond venation and apex pointed, with a few widely spaced proximally beyond venation then microtrichia more numerous in apical 0.4 of wing between the usual anterior and posterior rows (Fig. 19). Fore wing length 954, width 222, length/width 4.30, longest marginal setae 128. Hind wing length 872, width 47, longest marginal setae 118.

*Metasoma*. Ovipositor length 1300, 4.15 × as long as metatibia length (313) and extending anteriorly well forward of head (if it were attached and in its normal position) (Fig. 18).

**Male**. Unknown.

#### Derivation of species name.

The species is named after the continent of Africa, because this is the first species of *Paranaphoidea* reported from there.

### Allanagrus

Taxon classificationAnimaliaHymenopteraMymaridae

Noyes & Valentine, 1989

#### Type species.

*Allanagrus magniclava* Noyes & Valentine.

*Allanagrus* contains three described species in Australia (Lin et al. 2007) and one in New Zealand (Noyes & Valentine 1989), but the genus also occurs in the Oriental region (Triapitsyn 2014b). *Allanagrus* species have the clava 3-segmented (or apparently so in some doubtful cases) and tarsi 4-segmented, both features shared by at least one species of 18 other genera: *Allarescon* Noyes & Valentine, *Anaphes*, *Anneckia* Subba Rao, *Eustochus* Haliday, *Kompsomymar* Lin & Huber, *Krokella* Huber, *Neostethynium* Ogloblin, *Nesomymar* Valentine, *Nesopatasson* Valentine, *Notomymar* Doutt & Yoshimoto, *Paracmotemnus* Noyes & Valentine, *Paranaphoidea*, *Parastethynium* Lin & Huber, *Platystethynium* Ogloblin, *Polynemoidea* Girault, *Pseudanaphes* Noyes & Valentine, *Pseudocleruchus* Donev & Huber, and *Stethynium* Enock. These genera are not necessarily related, however. Three genera, *Nesomymar*, *Nesopatasson* and *Notomymar*, are only known from wingless specimens whose relationships are uncertain—these genera may be represented also by winged species described in other genera. The genera most similar to *Allanagrus* appear to be *Anneckia*, *Parastethynium* (both in Australasian region) and *Stethynium* (almost worldwide) based on: face with subantennal grooves, and frenum weakly sclerotized and apparently divided medially by a longitudinal groove. The strongly oblique suture of the compact clava in *Stethynium* removes this genus from further consideration here. The new species described below differs from *Anneckia* in that the campaniform sensilla of the dorsellum abut the anterior margin, as in one of the two species of *Parastethynium* (well separated from anterior margin in *Anneckia* and in the type species of *Parastethynium*) and the mandible (female) is well developed and probably has 2 distinct ventral teeth (not clearly visible in holotype of new species) and a dorsal, serrated edge somewhat as in *Parastethynium* (1 tooth in the reduced mandible of *Anneckia*). It differs from *Parastethynium* in that the fore wing is fairly narrow with rounded apex, and the hind wing is narrow (fore wing wide and apically truncate and hind wing quite wide in *Parastethynium*) and eye is much less setose (eye with many setae in *Parastethynium*). On balance of features we tentatively place the new species in *Allanagrus*. A detailed study of all these genera is needed to clarify their relationships and determine if some should be synonymized under others or, conversely, more should be proposed.

### Allanagrus occidentalis

Taxon classificationAnimaliaHymenopteraMymaridae

Huber & Triapitsyn sp. n.

http://zoobank.org/60090A76-341C-4DAE-B56D-6ECD7A2FC5CC

**Figures 25, 26.**
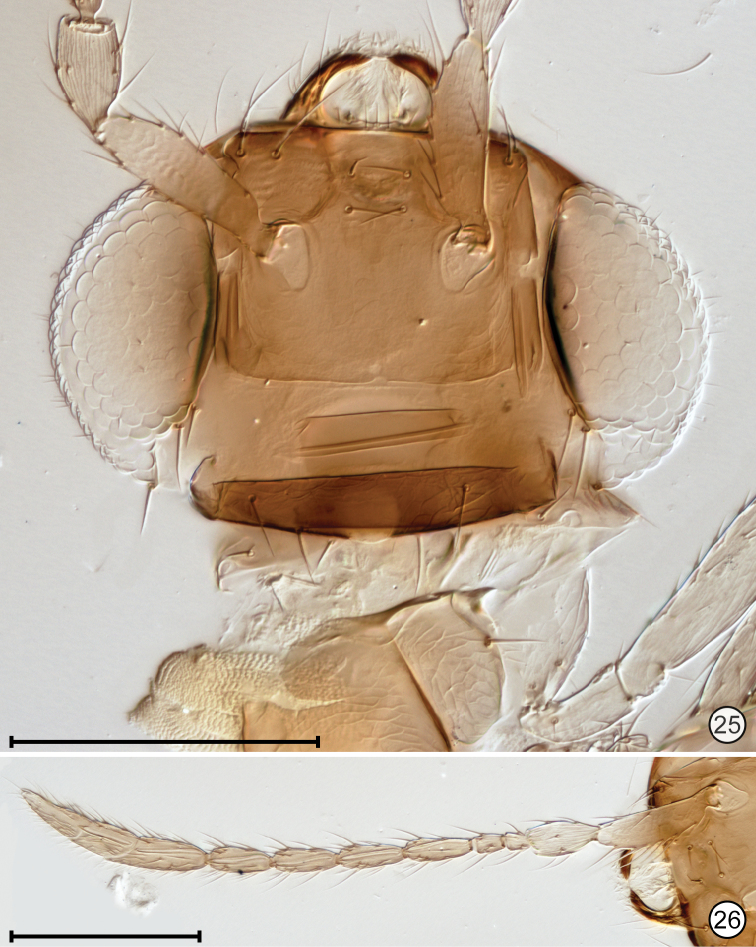
*Paranaphoidea occidentalis* holotype **25** head, anterior + crushed pronotum **26** antenna. Scale bars: 100 μm.

**Figures 27, 28.**
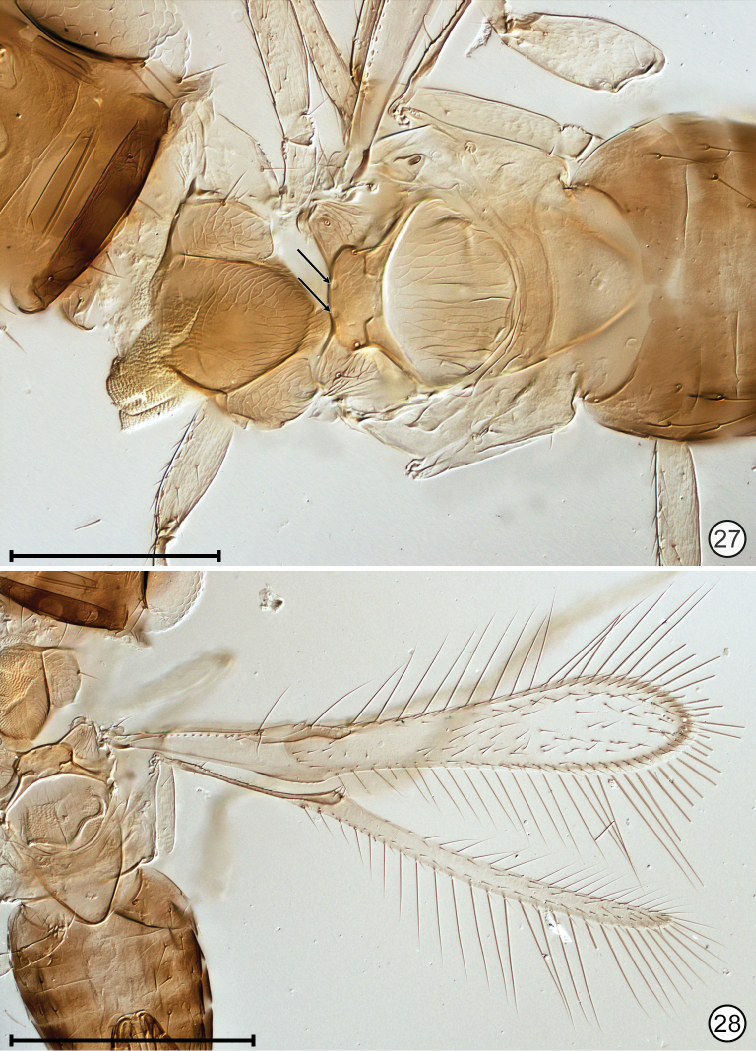
*Paranaphoidea occidentalis* holotype **27** mesosoma + base of metasoma **28** part of mesosoma and metasoma, and wings. Scale bars: 100 μm (**27**), 200 μm (**28**).

**Figures 29–31.**
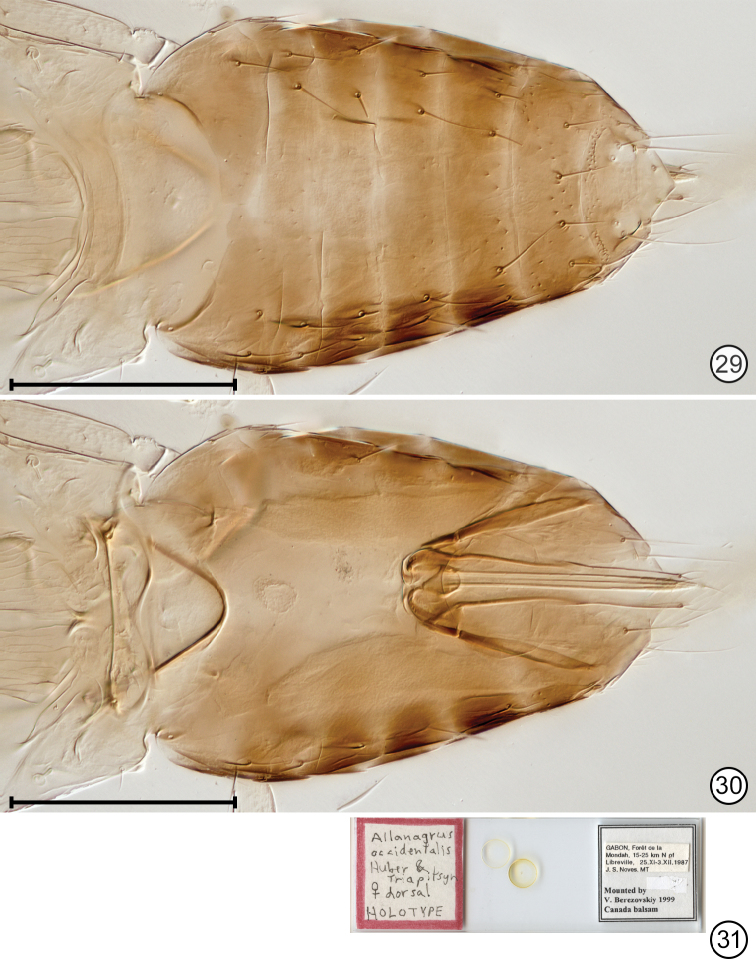
*Paranaphoidea occidentalis* holotype **29** apex of mesosma + metasoma, dorsal **30** apex of mesosma + metasoma as seen through body **31** holotype slide. Scale bars: 100 μm.

Figs 25–26 , 27, 28 , 29–31

#### Type material.

Holotype female (BMNH) on slide (Fig. 31) labelled: 1. “Allanagrus occidentalis Huber & Triapitsyn ♀ dorsal Holotype”. 2. “Gabon, Forêt de la Mondah, 15–25 km N of Libreville, 25.xi-3.xii.1987 J.S. Noyes. MT”. 3. “Mounted by V. Berezovskiy 1999 Canada balsam”.

#### Diagnosis.

Features that together distinguish *Allanagrus occidentalis* from other described species of *Allanagrus* (all of which occur in the Australian or Oriental regions) are: body small; fl_1_ and fl_2_ together shorter than either pedicel or fl_3_ (Fig. 26); and ovipositor shorter than metatibia and scarcely exserted beyond apex of gaster.

#### Description.

**Female.** Body length 450 (mesosoma + metasoma only). Head, midlobe of mesoscutum and metasoma brown; mesosoma mostly, antenna, and legs light brown or yellowish; wings mostly with faint brown suffusion except fore wing with oval hyaline area in about apical third.

*Head*. Head width 210. Subantennal groove ventral to each torulus present but apparently faint (Fig. 25). Mandible apparently with 2 distinct ventral teeth and a dorsal serrated edge.

*Antenna*. Funicle without mps on fl_1_ and fl_2,_ and with 1 mps on fl_3_–fl_6_ (Fig. 26); clava with at least 3 mps, apparently 1 on each of segments 1–3 (possibly more on each segment but clava mounted in dorsal view and not clearly visible). Scape with row of several setae along ventral margin. Measurements (length/width) of antennal segments: scape 68/18, pedicel 38/21, fl_1_ 15/9, fl_2_ 17/10, fl_3_ 37/13, fl_4_ 36/13, fl_5_ 34/13, fl_6_ 34/14, entire clava 100/≈18 (clava oriented in mostly dorsal view so appears narrow) with segments 1–3 (measured along dorsal margin) 39, 24, and 41, respectively. Length/width ratios of antennal segments: scape 3.84, pedicel 1.81, fl_1_ 1.65, fl_2_ 1.72, fl_3_ 2.91, fl_4_ 2.70, fl_5_ 2.61, fl_6_ 2.47, entire clava ≈5.53.

*Mesosoma*. Mesoscutum with oblique reticulate sculpture on lateral lobe and most of midlobe except posteromedially where sculpture is longitudinal. Scutellum with mainly oblique sculpture on anterior scutellum and with longitudinal reticulate sculpture on frenum. Dorsellum with campaniform sensilla in contact with anterior margin (Fig. 27, arrows indicate sensilla) and with a seta on each side at lateral margin.

*Wings*. Fore wing with microtrichia somewhat scattered, present behind parastigma and more evenly distributed in apical 0.4, but absent just beyond apex of venation and in the middle of the hyaline oval area (Fig. 28). Hind wing with a few microtrichia on surface near wing between the usual anterior and posterior rows. Fore wing length 463, width 75, length/width 6.16, longest marginal setae 146. Hind wing length 443, width 20, longest marginal setae 103.

*Metasoma*. Ovipositor length 134, 0.79 × as long as metatibia length (168) and barely exserted beyond apex of gaster (Figs 29, 30).

**Male**. Unknown.

#### Derivation of species name.

The species is the first *Allanagrus* reported from Africa and is named from Latin, *occidens -tis* meaning west, because it is by far the most westerly occurring species known for the genus.

## Supplementary Material

XML Treatment for Cleruchus

XML Treatment for Anaphes

XML Treatment for Anaphes quinquearticulatus

XML Treatment for Paranaphoidea

XML Treatment for Paranaphoidea (Idiocentrus) africana

XML Treatment for Allanagrus

XML Treatment for Allanagrus occidentalis
